# Author Correction: Bimodality and alternative equilibria do not help explain long-term patterns in shallow lake chlorophyll-a

**DOI:** 10.1038/s41467-023-36343-0

**Published:** 2023-02-01

**Authors:** Thomas A. Davidson, Carl D. Sayer, Erik Jeppesen, Martin Søndergaard, Torben L. Lauridsen, Liselotte S. Johansson, Ambroise Baker, Daniel Graeber

**Affiliations:** 1 Lake Ecology, Department of Ecoscience and Arctic Research Centre, Aarhus University, Aarhus, Denmark; 2 WATEC Aarhus University Centre for Water Technology, Aarhus University, Aarhus, Denmark; 3 Environmental Change Research Centre, Department of Geography, University College London, Gower Street, London, WC1E 6BT UK; 4 Sino-Danish Centre for Education and Research (SDC), University of Chinese Academy of Sciences, Beijing, China; 5 Limnology Laboratory, Department of Biological Sciences and Centre for Ecosystem Research and implementation, Middle East Technical University, Ankara, Turkey; 6 Institute of Marine Sciences, Middle East Technical University, Erdemli-Mersin, Turkey; 7 School of Health and Life Science, & National Horizons Centre, Teesside University, Middlesbrough, TS1 3BX UK; 8 Aquatic Ecosystem Analysis, Helmholtz-Centre for Environmental Research – UFZ, Brückstr. 3a, 39114 Magdeburg, Germany

**Keywords:** Ecosystem ecology, Limnology

Correction to: *Nature Communications* 10.1038/s41467-023-36043-9, published online 25 January 2023

In the original version of this Article, Table 1 contained several rounding errors. The correct version of Table 1 is:

Table 1. Number of lakes, means (1SD; minimum–maximum) of the contemporary data from the USA, Denmark and the full dataset.

**Table:** 

Variable	USA	Denmark	All data
Number of lakes	122	780	902
Mean depth (m)	2.0 (0.7; 0.4–3.0)	1.1 (0.7; 0.0–3.0)	1.2 (0.8; 0.0–3.0)
Area (ha)	150.4 (389.0; 1.7–3179.2)	34.2 (114.0; 0.0–1713.0)	49.9 (182.1; 0.0–3179.2)
Chlorophyll a (µg L^−1^)	37.0 (44.2; 0.3–362.5)	63.8 (65.5; 0.0–520.1)	57.3 (62.1; 0.0–520.1)
Total nitrogen (mg L^−1^)	1.4 (1.1; 0.1–5.9)	1.7 (1.0; 0.3–6.0)	1.6 (1.0; 0.1–6.0)
Total phosphorus (µg L^−1^)	87.8 (84.9; 4.6–516.9)	179.2 (194.0; 4.4–1457.9)	157.2 (178.3; 4.4–1457.9)

which replaces the previous incorrect version:

Table 1. Number of lakes, means (1SD; minimum–maximum) of the contemporary data from the USA, Denmark and the full dataset.

**Table:** 

Variable	USA	Denmark	All data
Number of lakes	122	780	902
Mean depth (m)	2.0 (0.7; 0.4–3.0)	1.1 (0.7; 0.01–3.0)	1.2 (0.8; 0.01 – 3.0)
Area (ha)	150.0 (389.0; 1.7–3179.0)	34.2 (114.0; 0.04–1713)	50.0 (182.0; 0.04–3179)
Chlorophyll a (µg L^−1^)	37 (44.2; 0.3–362.0)	63.8 (65.5; 0.1 – 520.0)	57.3 (62.1; 0.02–520.0)
Total nitrogen (mg L^−1^)	1.4 (1.1;0.1–5.9)	1.7 (1.0; 0.3–6.0)	1.7 (1.0; 0.14–6.0)
Total phosphorus (µg L^−1^)	90 (80; 0–520)	180 (190; 0–1460)	160 (180; 0–1460)

This has now been corrected in both the PDF and HTML versions of the Article.

