# Supplementary material for: Bimodality and alternative equilibria do not help explain long-term patterns in shallow lake chlorophyll-a
Source: Nat Commun. 2023 Jan 25;14:398. doi: 10.1038/s41467-023-36043-9 (PMC9873929; doi:10.1038/s41467-023-36043-9)
Supplement: Supplementary file 1 — Supplementary Information [file 41467_2023_36043_MOESM1_ESM.pdf]

Supplementary Information: Bimodality and alternative equilibria do not help explain long-term patterns in shallow lake chlorophyll-a

Thomas A. Davidson*[1&2], Carl D. Sayer[3], Erik Jeppesen[1, 2,4,5&6], Martin Søndergaard[1&4], Torben L. Lauridsen[1,2&4], Liselotte S. Johansson[1], Ambroise Baker[7] & Daniel Graeber*[8]

Supplementary Note 1. Assessment of alternative equilibria in long-term data.

We extracted all long-term time series (more than ten observed years) from the five-year mean dataset to examine multi-year mean approach for real data and to assess potential appearance of alternative stable states in the long-term data. All long-term data time series stem from the Danish part of the dataset, the US data consisted only of shorter time series. In total, 10 of the 99 lakes in the dataset with five-year means consisted of more than ten observed years. Supplementary Figure 1 shows how chlorophyll-a varied over decadal scales. The chlorophyll-concentrations of the time series are in different ranges. The time series are stationary or dynamic, and the kernel density plots indicate absence of bimodality in most cases, though in the absence of nutrient data this is neither evidence for or against alternative equilibria. Only the kernel density plots of the lakes “3000005”, 4200003”, and “4500005” exhibit bimodality to some extent over the course of the time series. This is, however, not evidence for alternative stable states, for this we need to examine the how TP and TN shape chlorophyll-a to see if there is more of a simple linear driver-response relationship.

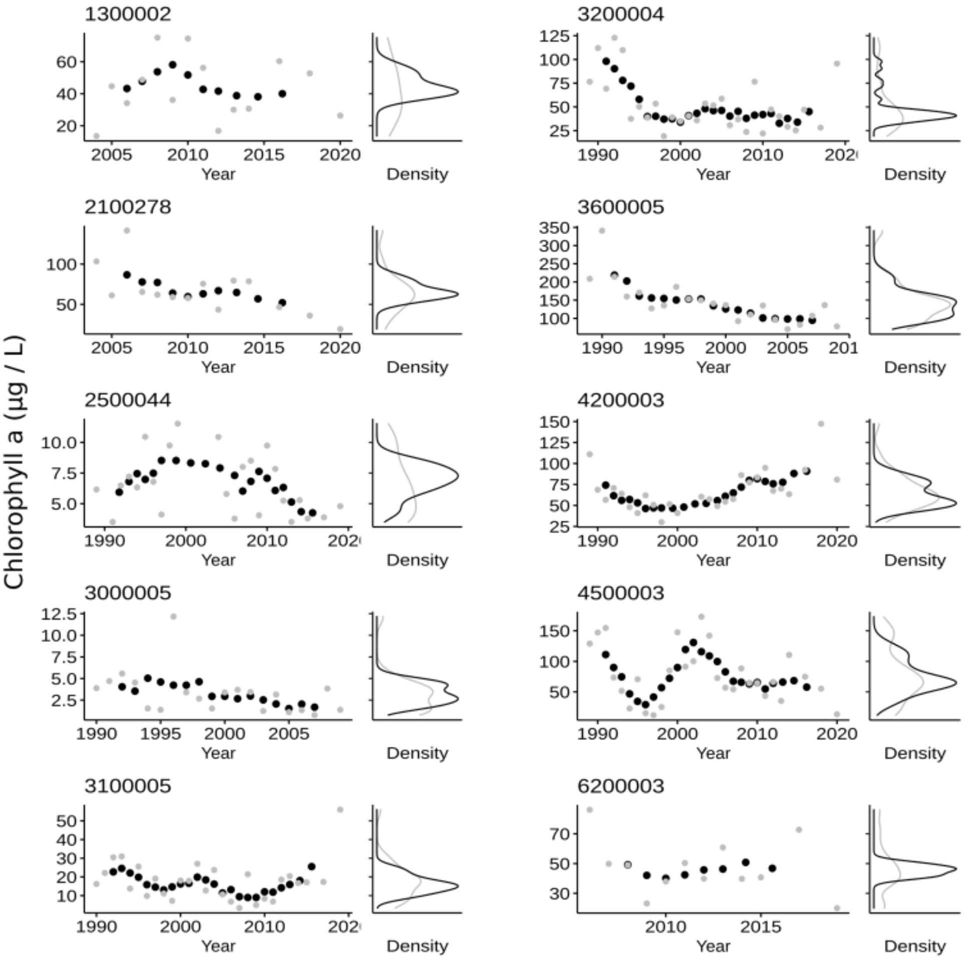

Supplementary Figure 1: Time series of real-world data. Grey dots are single-year observations (growing season means (May-September)), black dots are five-year rolling means. On the right hand side the kernel density plots of the single-year (grey) and five-year rolling means (black) are visible.

If we examine the relationship between both TP, TN and chlorophyll-a we can see that whilst some of the time series show some signs of bimodality in the Chl-a kernel density plots (Supplementary Figure. 1) the correlation between TP, TN and Chlorophyll-a are generally very linear, including the lakes "3000005" and "4500005". There are, however, some anomalous sites, such as lake "4200003" where there is very low chlorophyll-a for a given TP. This is, in fact, the same outlier dots visible in the TP – chlorophyll-a-a scatter plots of the main text (Fig. 2).

This different relationship between TP and chlorophyll-a could be argued to be a sign of alternative equilibria, however, a simpler explanation for this difference lies in the fact that the lake "4200003" is an extreme outlier in terms of the molar TN:TP ratio (Supplementary Figure. 2, bottom panel). Of all lakes with long-term data available, this lake is the only case with molar TN:TP ratios are well below Redfield ratio. This implies not TP but TN limitation of phytoplankton growth, which may explains its outlier status when only TP and Chlorophyll-a are considered (Supplementary Figure. 2 top-left panel). Recent discussions in the literature also propose common N limitation of phytoplankton at molar TN:TP ratios below 20 [1] . However, further in-depth analyses of the TN:TP ratio effects on the correlations between TN and / or TP and Chlorophyll-a are out of the scope of this paper.

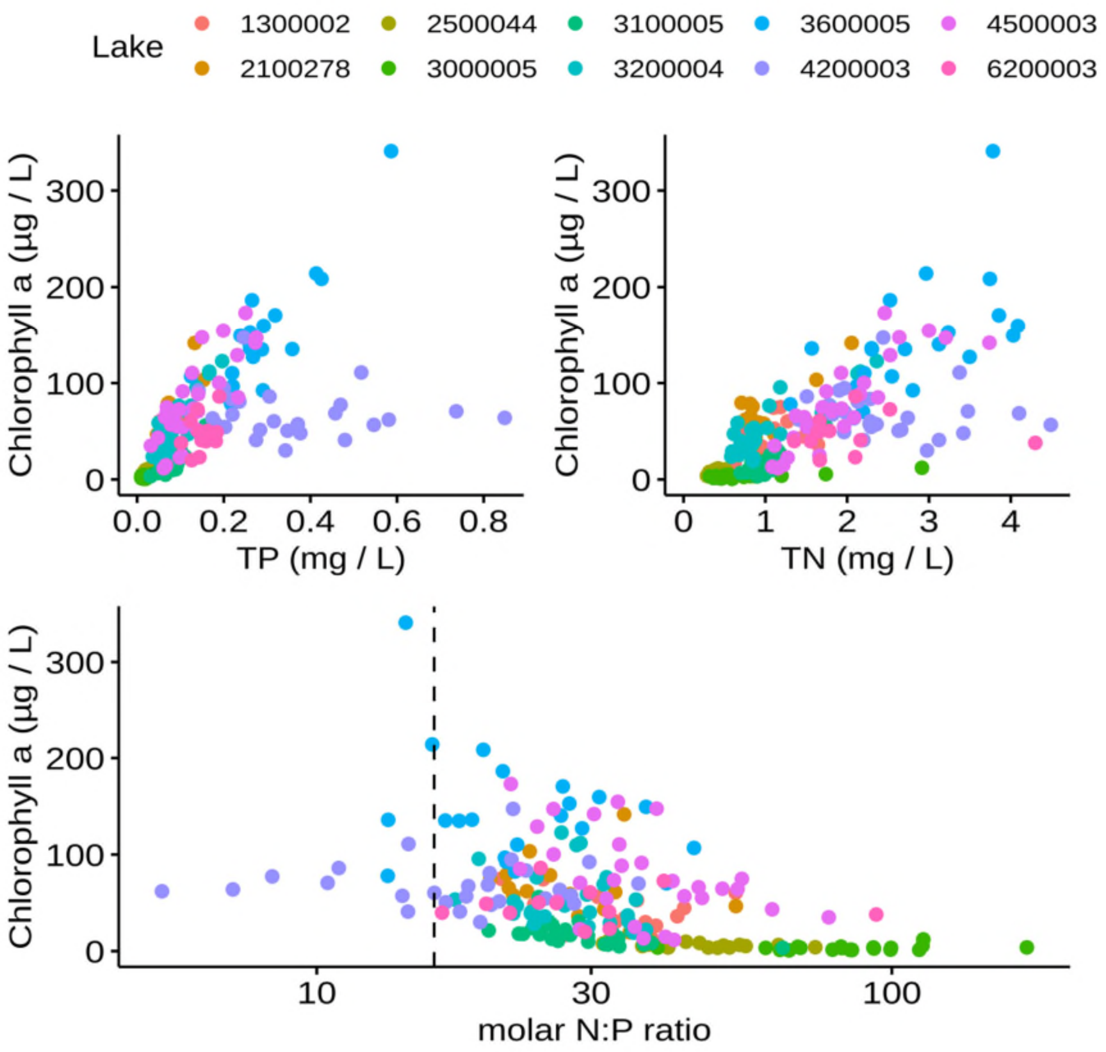

Supplementary Figure. 2: Scatter plots for TP (top-left) or TN (top-right) and Chl-a, and scatter plots for molar TN:TP ratios and Chl-a (bottom) for the five-year rolling means of the time series (Supplementary Figure. 1). Colours indicate the lakes.

Supplementary note 2. Description of simulation results on different possible stable-state constellations.

The simulation approach is described in the methods and is designed to assess the effects of the efficacy of the indicators used to identify the presence, absence or prevalence of the alternative stable states in simulated and real world data. The simulations were constructed to resemble real world data as much as possible, as such they are based on the true range of real nutrient data and the variability of the Chlorophyll-a response and for 99 lakes (five-year mean data consists of 99 lakes, see methods for details). We chose a constant number of years per lake (20 years per lake, which equals the maximum time series length of the real data) to better compare the different alternative-stable state scenarios, without adding the confounding factor of time-series length (variable time-series length is used in the scenarios in the main text to be as close to the real world data as possible). We used the following scenarios:

1. No alternative stable states (main text Fig.3 and Supplementary Figure. 3, left panel).
2. Unstable alternative states appearing randomly in a time series, with no long- term persistence (stability) of different states exists (Supplementary Figure. 3 right panel).
3. A scenario where alternative stable states appear at any point in the time series. To accommodate this the alternative stable state appeared at any time after the first 20% but before the last 20% of the time series. The 20% criterion prevents that this scenario partially consists of only five-year means of one alternative state, a constellation which is covered by scenario 4. (Supplementary Figure. 4 left panel)
4. Alternative stable states only appearing in different lakes, never in the same lake (main text Fig.3 and Supplementary Figure. 4, right panel).

The representative time series, the top panel of the plot, show how the five-year means remove much of the year-to-year variability in the data, smoothing the time series, and revealing that the average response of the y to a similar level of x is more or less stable (representing the Chl-a response to a stable nutrient concentration) (Supplementary Figure. 3, uppermost panels). Here the density distribution of the five-year means of the representative time series for the simulated dataset indicates unimodality of the y for the scenarios without alternative stable states and also for the scenario with unstable alternative states (Supplementary Figure. 3, right panel). The scatter plots indicate a decreasing variability around the linear model line with increasing years in the model, revealing the underlying linearity of the response of the changes in y (simulated Chl-a) to the changes in x (simulated nutrient). The proxies further corroborate this, where R^2 increases with higher numbers of mean years. Furthermore, we find a more unimodal distribution of model residuals and a unimodal distribution of kernel density plots with increasing numbers of years in the mean calculation. In combination, these proxies are diagnostic of the absence of alternative stable states in the simulated data. The reason for the higher R^2 and the unimodality of the residuals and the y response is the underlying linear relationship between the simulated x (nutrient) and y (Chl-a) over multi-year time scales, which is blurred by interannual short-term variability.

In contrast to a simple linear response of y to x (no alternative stable states), the unstable alternative simply increases the year-to-year variability of the response. This is reflected in a slightly lower R^2 compared with the

scenario where alternative stable states are absence and a slightly broader spread in the kernel density plot. However, due to the instability of the two states in this scenario, the y response still follows a linear pattern.

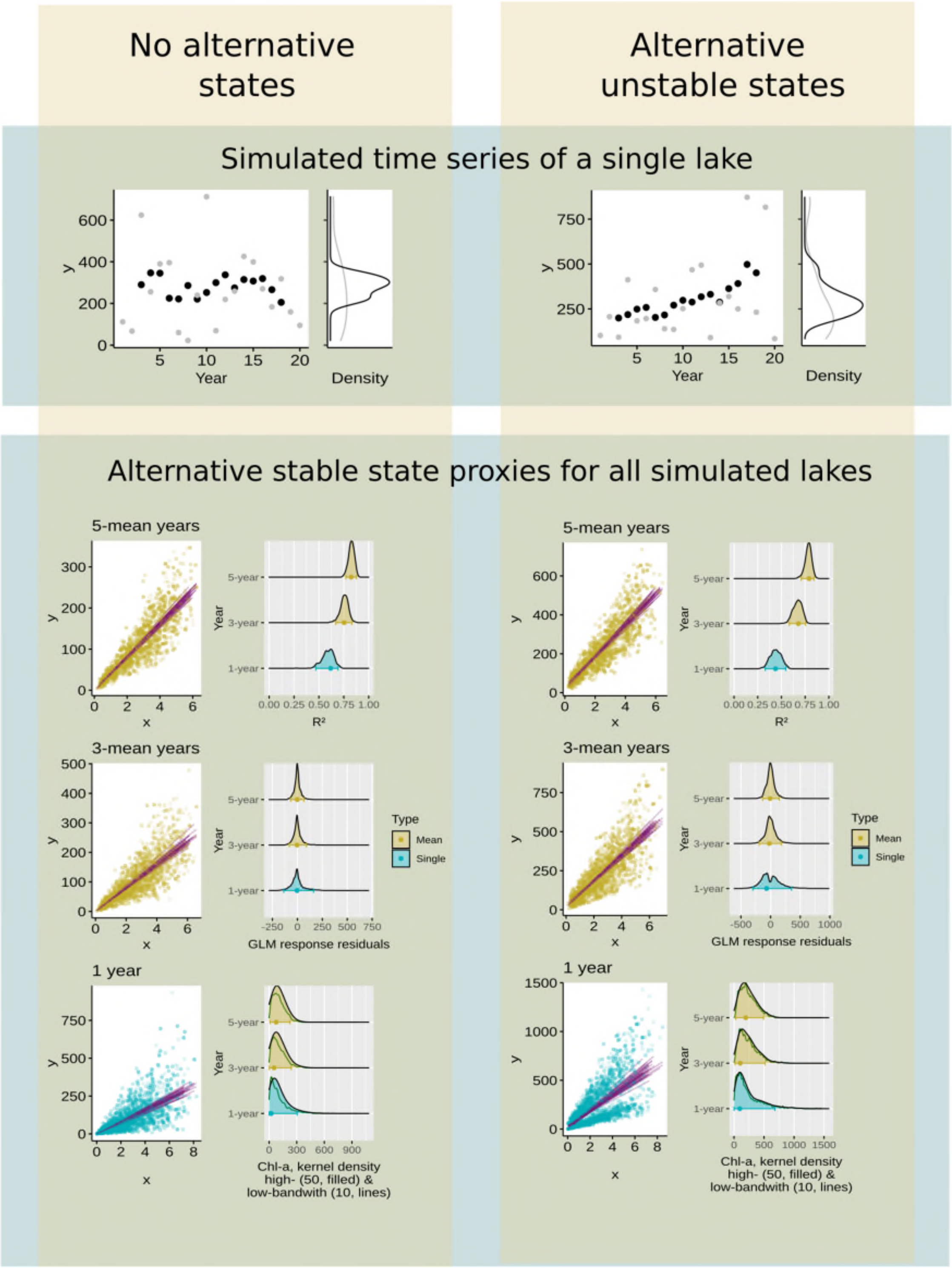

Supplementary Figure 3: Scenarios 1 (no alternative stable states) and 2 (unstable alternative states). Example of a single time series from the simulated dataset of 99 lakes (grey dots are single-year values & black dots are five-year rolling means) and proxies for the entire simulated dataset. The bootstrap procedure for each of the simulation scenarios ran with 500 iterations. The scatter plots contain only the first 120 iterations of the bootstrap procedure for better overview. The distributions of R^2, residuals and kernel density pots based on these multiple runs are shown as density plots with the error bars being the highest density interval with 95% credible interval.

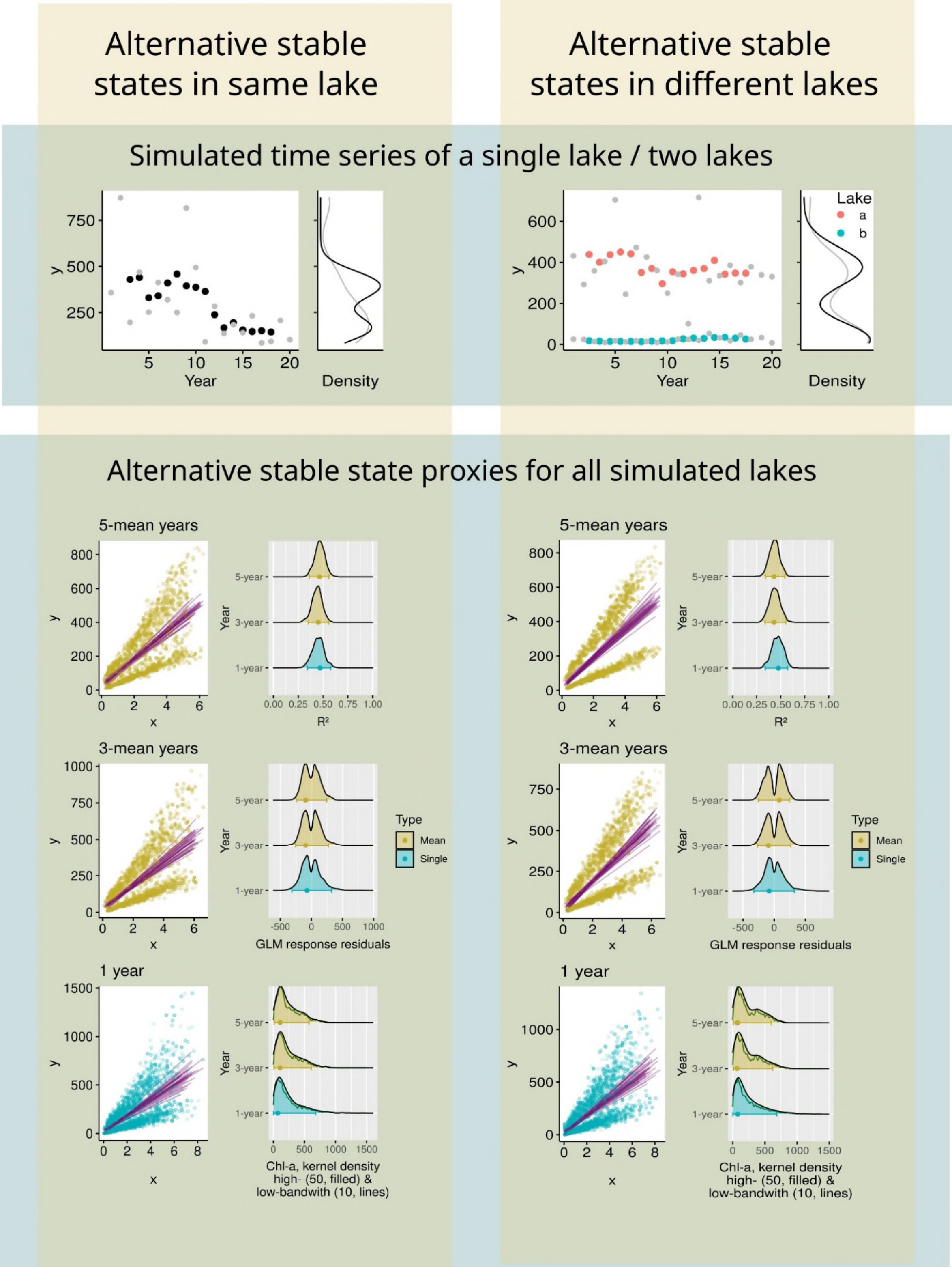

Supplementary Figure 4: Scenarios 3 (alternative stable states within one time series) and 4 (alternative stable states in time series from different lakes). Example of a single or two time series from the simulated dataset of 99 lakes (grey dots are single-year values & black dots are five-year rolling means) and proxies for the entire simulated dataset. The bootstrap procedure for each of the simulation scenarios ran with 500 iterations. The scatter plots contain only the first 120 iterations of the bootstrap procedure for better overview. The distributions of R^2, residuals and kernel density pots based on these multiple runs are shown as density plots with the error bars being the highest density interval with 95% credible interval.

For scenario 3 and 4, the example time series also show how the 5-year means smooth the year-to-year variation, making the underlying alternative stable states more visible within the time series of a single lake and in the comparison of two different lakes (Supplementary Figure. 4, uppermost panels). Here, the multimodality of the density distribution of the five-year means for y (representing two different, stable Chl-a responses to the same nutrient concentration) already suggest alternative stable states Supplementary Figure. 4, uppermost panels).

In contrast to the scenarios without alternative stable states, the scatter plots reveal a clear two-level response of y (simulated Chlorophyll-a) response to the same x (simulated nutrient concentration) with a higher number of mean years. Furthermore, the R^2 of the GLMs do not increase as the number of years represented in the means increases, reflecting the two distinct linear responses of y (chlorophyll-a-a) to the x (nutrient). Finally, the residuals of the GLMs reveal clear multimodality and the high-bandwidth kernel density plots do not converge to a normal distribution with a higher number of mean years, further corroborating the R^2 proxy and scatter plots.

Thus, the use of this gradient of simulations containing different degrees of alternative equilibria show that the diagnostics of the R^2, residual pattern and kernel density plot have patterns that are characteristic of both the presence, absence and even prevalence of alternative equilibria in the data. Thus, the combination of proxies can be used to test both the presence and absence of alternative stable states in real world data.

Combining data from different lakes with our bootstrap approach results in better detection of alternative stable states than traditional approaches

In combination with the proxies, the hierarchical bootstrap approach has another advantage. As shown in scenario 4, with our approach we find alternative stable states even if the alternative stable states appear in the time series of different lakes. Such a scenario is especially likely for short time series, where the entire time series may only be in one alternative state. We often have short time series in the data (in the dataset with five-year means, most of the time series (89 out of 99) are ten years long or even shorter, see also the analysis of long-term real data below). Hence, our approach will detect alternative stable states in cases where an independent investigation of time series from single lakes might fail, which makes it a powerful means to detect both the presence and absence of stable states in multi lake data sets of variable length.

Supplementary note 3: Alternative stable state assessment for real data with limited data range.

The existence of alternative equilibria in shallow lakes is generally considered possible within a limited range of nutrient enrichment. Thus, it could be that the alternative stable states present in the data are obscured when the full dataset is analysed. In order to check this we repeated the analysis on a more limited TN and TP concentration range. We filtered and re-analyzed the data, only keeping data points within the following two ranges: - TN concentration = 0.5 - 2 mg / L - TP concentration = 0.05 - 0.4 mg / L.

In the filtered data, 1329 out of the original 2876 single-year data points, 289 out of 1028 three-year mean data points and 212 out of the 864 five-mean year data points remained. The filtered data consisted of data points from 550, 48 and 27 lakes for the single-year data, three-year means and five-year means, respectively.

The smaller range resulted in lower R^2 of the models, but the patterns revealed by the multi-year means result in higher R^2 compared with single-year data was largely consistent. The exception being the five-year mean TN models for which both, the single-year and mean data resulted in very low R^2 (Supplementary Figure. 5). This can be explained by the fact that at the restricted nutrient range, TP exerts more influence on chlorophyll-a than TN and this becomes more evident at the multi-year scale. This difference in influence of nutrients over different portions of the enrichment gradient has been demonstrated by recent work using boosted regression trees on the Danish part of the data set [2], where the predictive power of TP of chlorophyll a was highest in the low range of TP enrichment whereas TN becomes more important at higher levels of enrichment. The filtering of the data to have reduced range of enrichment cut out the portions of the gradient where the influence of TN is greatest. Due to the lower number of samples, the errors of all proxies were higher, reducing the clarity of the results compared with the full data set. Still, we see no indication of alternative stable states in the scatter plots (two groups of dots are not appearing, Supplementary Figure 5), the Kernel density plots or model residuals (Supplementary Figure 6) (no signs of bimodality in residuals or Kernel density plots). In contrast, the indicators of R^2 and residual patterns for the TP and combined TP and TN models align closely with the patters expected in the absence of alternative equilibria in the data.

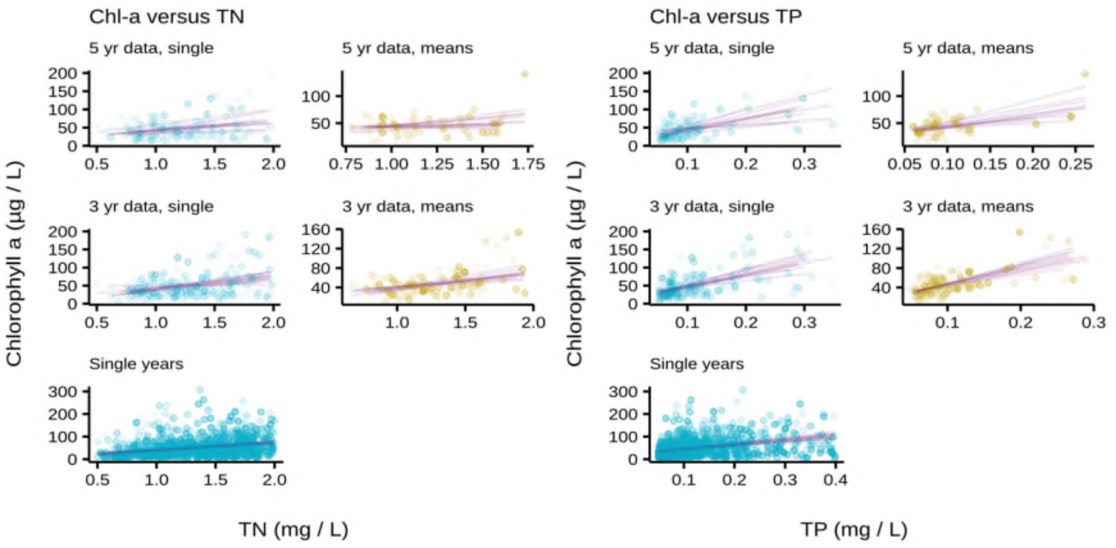

Supplementary Figure 5. Scatter plots for data with limited concentration range. 1000 random samples were run per bootstrap analysis, for each of which we calculated GLM models. The final numbers vary slightly as the GLM models did not always converge. The variation in the slope is a reflection of the multiple runs.

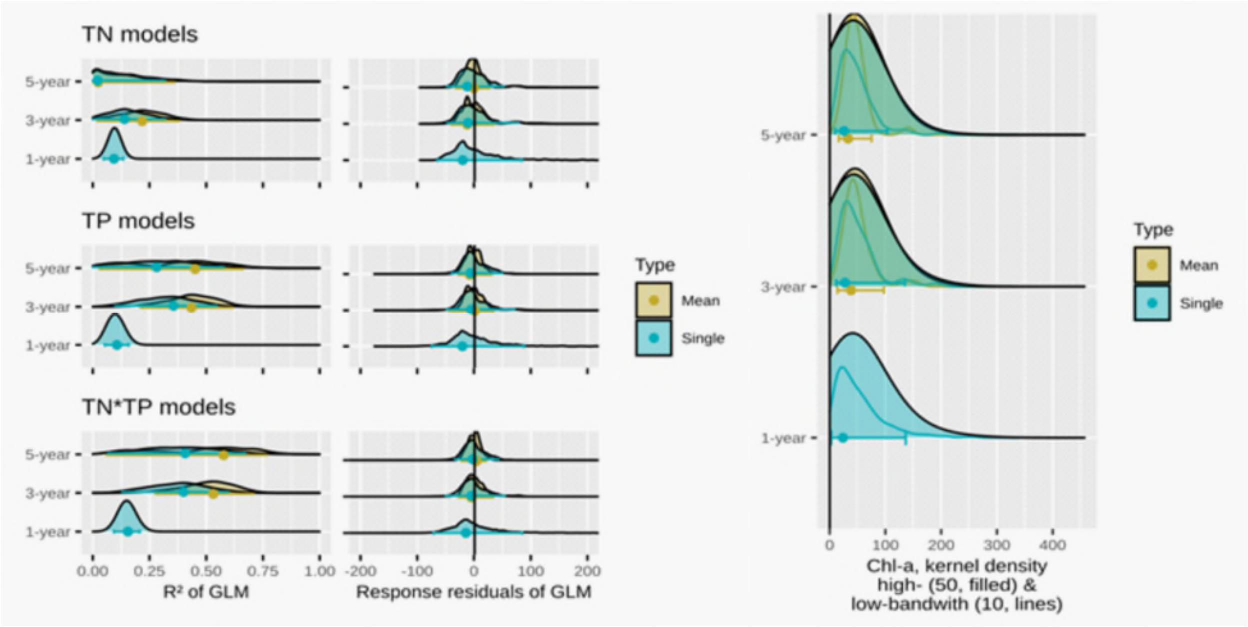

Supplementary Figure 6. Diagnostic indicator plots for data with limited concentration range. The distributions of R^2, residuals and kernel density plots based on these multiple runs are shown as density plots with the error bars being the highest density interval with 95% credible interval.

Supplementary note 4: Results from analysis of the lakes from different countries separately.

Results of the analysis from only Danish lakes (Supplementary Figure. 7) showing results of the diagnostic tests i) change in R^2 ii) patterns in models residuals and iii) Kernel density plot as the temporal scale increases (single year, three year and five year means) this data follows the patterns of the simulated data set where alternative equilibria are not present. The R^2 of the models increase in their explanatory power, though the multi-year mean data only reliably explains more variance than its single year equivalent for TN and there is no bimodality in the residual of the models.

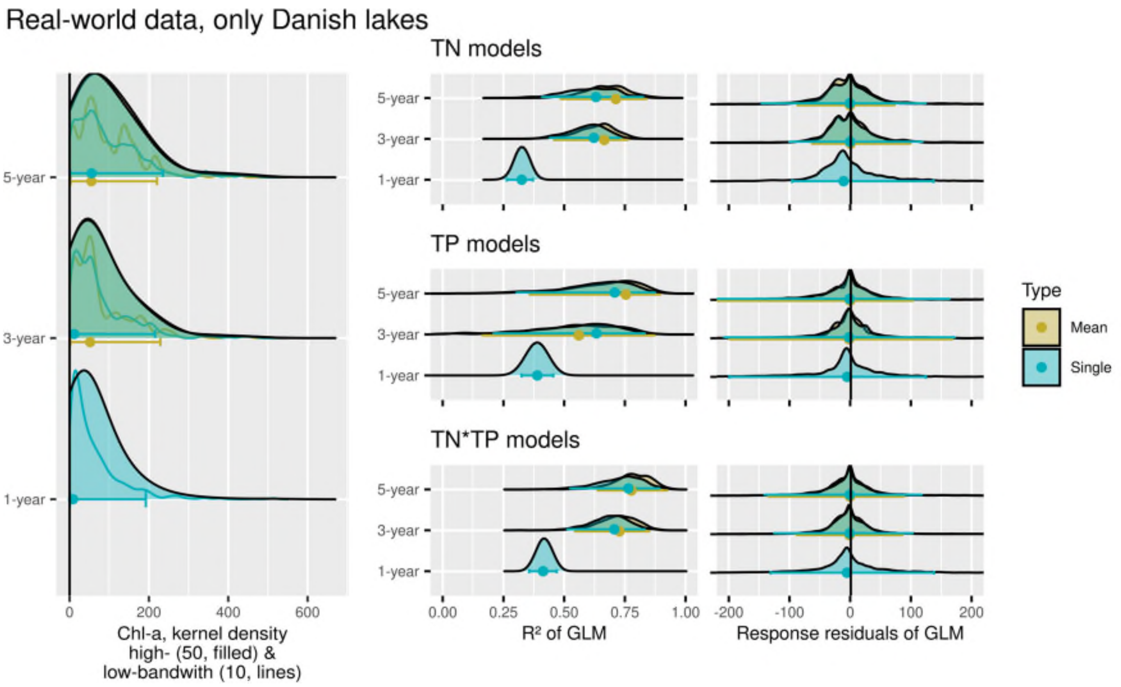

Supplementary Figure 7: Results of the analysis from only Danish lakes showing results of the diagnostic tests i) Kernel density plots, ii) change in R^2 and iii) patterns in models residuals as the temporal scale increases (single year, three

year and five year means) this data follows the patterns of the simulated data set with no alternative equilibria, as the Kernel density plots indicate no bimodality, the R^2 of the models increase in their explanatory power, though the multi-year mean data only reliably explains more variance than its single year equivalent for TN and there is no bimodality in the residual of the models. The distributions of R^2, residuals and kernel density plots based on these multiple runs are shown as density plots with the error bars being the highest density interval with 95% credible interval.

Results of the analysis from only U.S. lakes (Supplementary Figure. 8) shows results of the diagnostic tests i) R^2 ii) patterns in models residuals and iii) shape of the kernel density plot the temporal scale increases (single year, three year and five year means). These data follow the patterns of the simulated data set with no alternative equilibria, as the R^2 of the models increase in their explanatory power and the multi-year means always explain more variance than their single year equivalent and there is no bimodality in the residual of the models or in the kernel density function plot of chlorophyll-a.

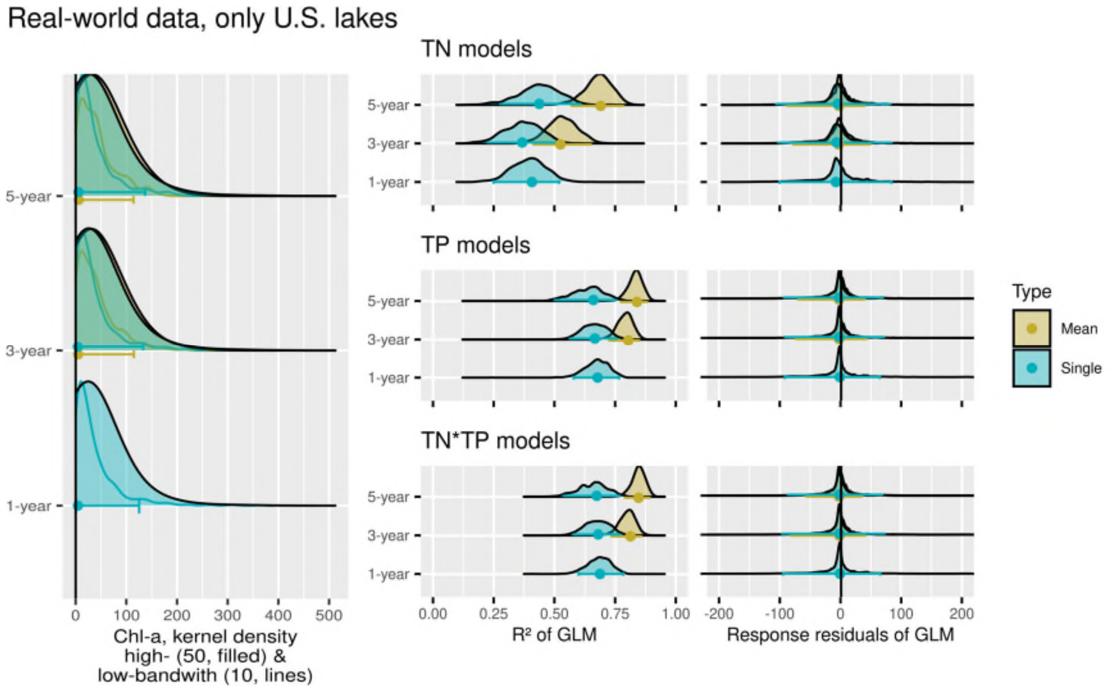

Supplementary Figure 8: Results of the analysis from only U.S. lakes showing results of the diagnostic tests i) Kernel density plots, ii) change in R^2 and iii) patterns in models residuals as the temporal scale increases (single year, three year and five year means) this data follows the patterns of the simulated data set with no alternative equilibria, as the Kernel density plots indicate no bimodality, the R^2 of the models increase in their explanatory power, though the multi-year mean data only reliably explains more variance than its single year equivalent for TN and there is no bimodality in the residual of the models. The distributions of R^2, residuals and kernel density plots based on these multiple runs are shown as density plots with the error bars being the highest density interval with 95% credible interval.

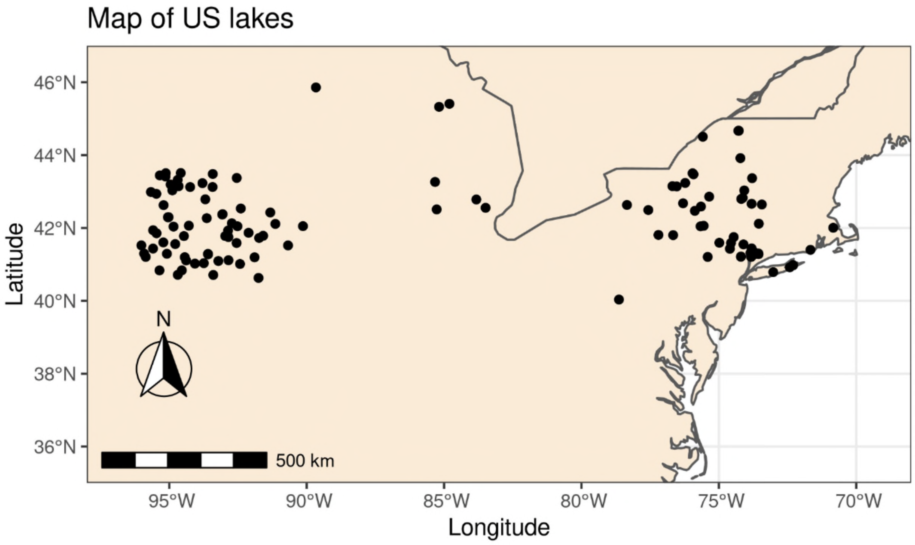

Supplementary Figure 9. Location of sites from the LAGOSNE database

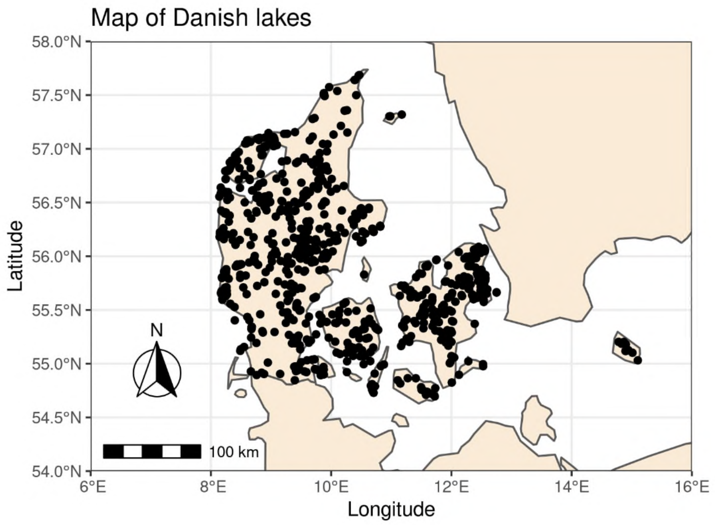

Supplementary Figure 10. Location of sites from the Danish database (odaforalle).

Supplementary References

1. Paerl HW*, et al.* It takes two to tango: when and where dual nutrient (N & P) reductions are needed to protect lakes and downstream ecosystems. *Environ. Sci. Technol.* **50**, 10805-10813 (2016).

2. Søndergaard M, Davidson TA, Lauridsen TL, Johansson LS, Jeppesen E. Submerged macrophytes in Danish lakes: impact of morphological and chemical factors on abundance and species richness. *Hydrobiologia*, (2021).

Supplementary code

R scripts for data preparation and analysis are available at

https://git.ufz.de/graeber/alternative-stable-states-do-not-stand-the-test-of-time

1. data preparation, US data – LAGOSNE.R

2. data preparation, Danish data.R
3. Combination of datasets, selection of lakes of mean calculation, calculation of bootstrapped data for combined data sets.R
4. R code for data simulation
