# Supplementary material for: Bimodality and alternative equilibria do not help explain long-term patterns in shallow lake chlorophyll-a
Source: Nat Commun. 2023 Jan 25;14:398. doi: 10.1038/s41467-023-36043-9 (PMC9873929; doi:10.1038/s41467-023-36043-9)
Supplement: Supplementary file 3 — Reporting Summary [file 41467_2023_36043_MOESM3_ESM.pdf]

nature portfolio

Corresponding author(s): Thomas Davidson & Daniel Graeber

Last updated by author(s): Jan 4, 2023

Reporting Summary

Nature Portfolio wishes to improve the reproducibility of the work that we publish. This form provides structure for consistency and transparency in reporting. For further information on Nature Portfolio policies, see our Editorial Policies and the Editorial Policy Checklist.

Statistics

For all statistical analyses, confirm that the following items are present in the figure legend, table legend, main text, or Methods section.

n/a	Confirmed	
	x	The exact sample size (n) for each experimental group/condition, given as a discrete number and unit of measurement
	x	A statement on whether measurements were taken from distinct samples or whether the same sample was measured repeatedly
x		The statistical test(s) used AND whether they are one- or two-sided<br>*Only common tests should be described solely by name; describe more complex techniques in the Methods section.*
	x	A description of all covariates tested
	x	A description of any assumptions or corrections, such as tests of normality and adjustment for multiple comparisons
	x	A full description of the statistical parameters including central tendency (e.g. means) or other basic estimates (e.g. regression coefficient) AND variation (e.g. standard deviation) or associated estimates of uncertainty (e.g. confidence intervals)
x		For null hypothesis testing, the test statistic (e.g. F, t, r) with confidence intervals, effect sizes, degrees of freedom and P value noted<br>*Give P values as exact values whenever suitable.*
x		For Bayesian analysis, information on the choice of priors and Markov chain Monte Carlo settings
x		For hierarchical and complex designs, identification of the appropriate level for tests and full reporting of outcomes
x		Estimates of effect sizes (e.g. Cohen's d, Pearson's r), indicating how they were calculated

Our web collection on statistics for biologists contains articles on many of the points above.

Software and code

Policy information about availability of computer code

Data collection	Data was collected from two open access databases: LAGOSNE database - using the R LAGOSNE package version 2.0.2 and from the Danish ODA database- https://odaforalle.au.dk/main.aspx
Data analysis	All analysis, data preparation stage, bootstrapping procedure and analysis was carried out in R version 4.2.1. Code can be accessed in Git Lab- https://git.ufz.de/graeber/alternative-stable-states-do-not-stand-the-test-of-time. The link to the Git Lab site is also provided in the supplementary materials. In addition a report on the data simulations carried out in R is provided in supplementary information and is available on GitLab.

For manuscripts utilizing custom algorithms or software that are central to the research but not yet described in published literature, software must be made available to editors and reviewers. We strongly encourage code deposition in a community repository (e.g. GitHub). See the Nature Portfolio guidelines for submitting code & software for further information.

Data

Policy information about availability of data

All manuscripts must include a data availability statement. This statement should provide the following information, where applicable:

- Accession codes, unique identifiers, or web links for publicly available datasets
- A description of any restrictions on data availability
- For clinical datasets or third party data, please ensure that the statement adheres to our policy

The data are publicly available in the LAGOSNE database (https://lagoslakes.org/lagos-ne/) and the ODA database https:// odaforalle.au.dk/main.aspx.

Human research participants

Policy information about studies involving human research participants and Sex and Gender in Research.

Reporting on sex and gender	N/A
Population characteristics	N/A
Recruitment	N/A
Ethics oversight	N/A

Note that full information on the approval of the study protocol must also be provided in the manuscript.

Field-specific reporting

Please select the one below that is the best fit for your research. If you are not sure, read the appropriate sections before making your selection.

☐ Life sciences ☐ Behavioural & social sciences ☒ Ecological, evolutionary & environmental sciences

For a reference copy of the document with all sections, see nature.com/documents/nr-reporting-summary-flat.pdf

Ecological, evolutionary & environmental sciences study design

All studies must disclose on these points even when the disclosure is negative.

Study description	The study combined two large datasets on shallow lakes from the USA and Denmark (with a total of over 900 lakes) to investigate how the relationship between nutrient enrichment and chlorophyll-a changes as the number of years included in the analysis increases. In oder to assess the veracity of the theory of alternative stable states in shallow lakes we compared the response of simulated data (with and without alternative equilibria) with the response of real world data as the number of years in the analysis increased.
Research sample	We used data on lakes under 3 metre average depth where multiple years of observations existed. There were from the USA https:// lagoslakes.org/lagos-ne/) and Denmark https://odaforalle.au.dk/main.aspx . The data sets contained just over 900 lakes which had at least a single years observation of chlorophyll-a, TP and TP which was based on at least 3 samples taken over the growing season.
Sampling strategy	To provide a robust approach with which to compare single years observations with multiple years means of data (i.e 2 year means of chlrophyll-a, TP and TN - up to 6 year means where the n was 99) we used a hierachical bootstrapping approach which was appropriate of the potentially nested or time linked data. For temporal perspective, for example the 2 year means, data were randomly selected from all the possible permutations of 2 year means - with an n of the number of lakes that could have 2 year means (203. see table 2). This random selection was carried out 1000 times and GLM models and other diagnostic tests carried out each time in order to quantify the variability of response within the datasets. The single year comparison from each temporal perspective was done on a single years data from the same lakes selected in each of the 1000 random iterations.
Data collection	These are described in the respective databases.
Timing and spatial scale	The data from the database span from the 1980s to the present.
Data exclusions	Data were excluded based on depth so as to focus on shallow lakes, using a well established cut off of average depth of 3 m to define shallow lakes.
Reproducibility	This is not an experimental study, but providing the R codes for the data preparation and analysis mean the process is entirely transparent and reproducible.
Randomization	N/A

Blinding	N/A blinding for group allocation in this study is not possible as the data were pre-collected and the only grouping was the geographical origin of the sites. The randomised bootstrapping procedure used in the study is was blind to this grouping.

Did the study involve field work? ☐ Yes ☒ No

Reporting for specific materials, systems and methods

We require information from authors about some types of materials, experimental systems and methods used in many studies. Here, indicate whether each material, system or method listed is relevant to your study. If you are not sure if a list item applies to your research, read the appropriate section before selecting a response.

Materials & experimental systems

n/a	Involved in the study
☒	☐ Antibodies
☒	☐ Eukaryotic cell lines
☒	☐ Palaeontology and archaeology
☒	☐ Animals and other organisms
☒	☐ Clinical data
☒	☐ Dual use research of concern

Methods

n/a	Involved in the study
☒	☐ ChIP-seq
☒	☐ Flow cytometry
☒	☐ MRI-based neuroimaging
